# Can the preterm lung recover from perinatal stress?

**DOI:** 10.1186/s40348-016-0043-9

**Published:** 2016-04-13

**Authors:** Matthias C. Hütten, Tim G. A. M. Wolfs, Boris W. Kramer

**Affiliations:** Neonatology, Department of Pediatrics, Maastricht University Medical Center, Maastricht, Netherlands; Neonatology, Department of Pediatrics, Aachen University Hospital, Aachen, Germany; Neonatology, Department of Pediatrics, Würzburg University Hospital, Würzburg, Germany; Faculty of Health, Medicine and Lifesciences, School for Oncology and Developmental Biology, Maastricht University, Maastricht, The Netherlands

**Keywords:** Lung injury, Chorioamnionitis, Glucocorticosteroids, Caffeine, Oxygen toxicity, Ventilation, Bronchopulmonary dysplasia, Vitamin A

## Abstract

After birth, adequate lung function is necessary for the successful adaptation of a preterm baby. Both prenatal and postnatal insults and therapeutic interventions have an immediate effect on lung function and gas exchange but also interfere with fetal and neonatal lung development. Prenatal insults like chorioamnionitis and prenatal interventions like maternal glucocorticosteroids interact but might also determine the preterm baby’s lung response to postnatal interventions (“second hit”) like supplementation of oxygen and drug therapy. We review current experimental and clinical findings on the influence of different perinatal factors on preterm lung development and discuss how well-established interventions in neonatal care might be adapted to attenuate postnatal lung injury.

## Introduction

**Fig. 1:**
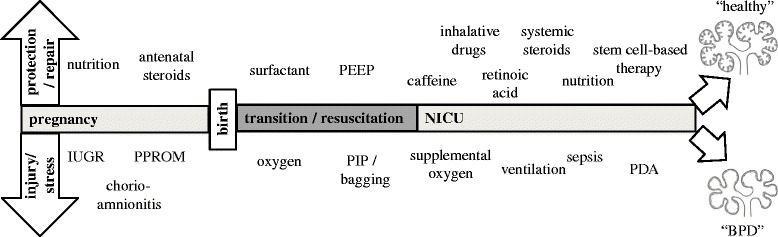
Multiple factors influence lung development in preterm infants. Postnatally, both potentially protective and injurious factors are mainly associated with therapeutic means. *IUGR* intra-uterine growth restriction, *PPROM* preterm premature rupture of membranes, *PIP* positive inspiratory pressure, *PEEP* positive end-expiratory pressure, *PDA* persistent ductus arteriosus, *NICU* neonatal intensive care unit, *BPD* bronchopulmonary dysplasia

The lung function of a preterm baby is key to the successful adaptation after birth since no gas exchange via diffusion will be possible without sufficient maturity of the alveolar and capillary unit [1]. The development of the fetal lung is affected by antenatal maternal glucocorticoids, chorioamnionitis, and maternal nutrition [2]. Altered fetal development affects pulmonary responses after birth to subsequent—postnatal—injuries such as oxygen toxicity or responses to drugs [3]. The different effects of antenatal and postnatal insults and interventions are summarized in Fig. 1. In this review, we will give a concise overview of recent developments on lung function and growth that highlight the interaction between factors that determine lung plasticity in the context of lung injury, regeneration, and immunomodulation and in the development of bronchopulmonary dysplasia (BPD).

## Chorioamnionitis as prenatal insult

The exposure to microbes in utero appears to be very common in preterm deliveries [4]. Chorioamnionitis induced by different microbial triggers results in pulmonary inflammation and subsequent structural simplification in the alveoli and vasculature of the fetal lung [5, 6]. In the clinical course of postnatal pulmonary adaptation and development in preterm infants, chorioamnionitis plays a dual role. On the one hand, exposure to chorioamnionitis might protect preterm infants from respiratory distress syndrome (RDS) [7]. In animal models, prenatal exposure to inflammatory stimuli supported surfactant production and structural maturation and resulted in better lung compliance [8]. However, surfactant replacement therapy has been shown to be less effective in preterm infants who were exposed to chorioamnionitis and developed a fetal inflammatory response [9]. Moreover, data from experimental models show that lung injury after exposure to intrauterine inflammation depends among others on the type of the infectious agents and the time of onset of intrauterine inflammation [3]. This might explain the inconsistent effect of chorioamnionitis on postnatal pulmonary adaptation. On the other hand, growing evidence suggests an important role of intrauterine inflammation as contributing factor to the development of BPD [10–12]. Animal experiments revealed that intrauterine inflammation resulted in structural lung impairment [13] and disturbance of developmental pathways in the lung, impairing growth factors and branching morphogenesis [6]. These long-term effects might also depend on the severity of the inflammatory response. In a recent clinical study, histological severity of fetal inflammation in cases of chorioamnionitis was independently associated with development of BPD, even after adjusting for gestational age [14]. Moreover, preexposure to chorioamnionitis alters inflammatory reaction on a second inflammatory stimulus [15]. Fetal attenuated reaction on repeated inflammatory stimuli can prevent lung injury [16]; therefore, the association between prenatal inflammation and postnatal lung injury remains complex.

## Chorioamnionitis and antenatal corticosteroids—combined effects

The course and the time point of onset of infection are—in cases of clinically silent chorioamnionitis—not to be determined [17]. In cases of clinical chorioamnionitis, the maternal symptoms suggest the onset of a maternal response to microbes which does not preclude the use of antenatal maternal steroids [18]. Antenatal maternal corticosteroid therapy accelerates fetal lung maturation [19] and supports endogenous surfactant production [20]. Although there is an ongoing discussion about the ideal preparation and dosing [21], maternal glucocorticosteroids are the gold standard treatment when premature delivery is expected [22]. However, experimental data revealed that the combined effects of prenatal exposure to chorioamnionitis and glucocorticosteroids are variable and do not simply “add up". The time point of administration of antenatal steroids before or after the onset of chorioamnionitis in a sheep model of LPS-induced chorioamnionitis was studied in order to assess the effects of lung maturation and immune modulation in a preclinical model [23]. Inhibition or even prevention of impaired structural pulmonary development appeared to be dependent on the timing of administration of maternal steroids [24, 25]. Administration before onset of LPS-induced chorioamnionitis reduced pulmonary inflammation [25], counteracted LPS-induced transforming growth factor β (TGFβ) pathway activation [23], and prevented structural changes [24]. Pulmonary inflammation was not attenuated if administration of maternal glucocorticoids was done after onset of chorioamnionitis, and inflammatory cells in the lung increased [25]. In contrast, positive effects of maternal glucocorticoids on lung function and surfactant metabolism were enhanced when they were given after onset of pulmonary inflammation [25]. These findings emphasize that the mechanisms linking intrauterine inflammation to the induction of lung structural changes are multi-factorial [6].

One possible link is oxidative stress, with BPD being considered as an oxygen-radical disease of the preterm [26]. Chorioamnionitis has multiple effects on levels of reactive oxygen species and enzymes involved in the detoxification of reactive oxygen species. However, these effects are not invariably positive or negative. Data obtained in the preclinical lamb model of chorioamnionitis shows that acute intrauterine inflammation precedes increases in oxidants in the fetal airways [27] but also increases in antioxidant enzyme activity in fetal lung tissue [28]. Taken together, the effect of chorioamnionitis on oxidative stress in the lung still needs to be elucidated. In addition, it is unclear whether chorioamnionitis leads to antenatal conditioning of fetal redox systems which may affect the response to a pre- or postnatal second hit [29–31]. For example, modulation of fetal oxidative stress has been reported after maternal glucocorticoid administration in both experimental [32, 33] and clinical settings [34–36], but it remains unclear if these effects vary depending on the presence or absence of inflammation. Moreover, inflammation can also result from oxidative stress [37], which highlights the role of oxygen toxicity as risk factor for adverse neonatal outcomes [31].

## Postnatal interventions—oxygen

The fetus develops in a low oxygen environment, and the arterial partial pressure of oxygen (PaO_2_) physiologically rises directly after birth [38]. This abrupt change in oxygen content of blood and tissue may induce physiological maturation of metabolic processes after birth [39]. However, an excess supply of oxygen resulting in hyperoxia might have detrimental effects on infants born prematurely. Oxygen supplementation is one of the most common therapeutic interventions in resuscitation of newborns [40]. However, its historically generous use in the delivery room has been abandoned in the last years due to new evidence from clinical studies [41]. In the ground breaking Resair 2 study by Saugstad et al., the authors showed that resuscitation of term babies after asphyxia could efficiently be performed with room air instead of 100 % oxygen [42].

In preterm infants, current guidelines advocate the use of a mixture of air and oxygen according to the infants’ oxygen saturation (SpO_2_). These are based on the observation that an increase in oxygenation after birth is a gradual process [43]. A recent meta-analysis of studies comparing different initial fractions of oxygen (FiO_2_) in delivery room stabilization and resuscitation of preterm infants ≤32 weeks showed a trend towards a lower mortality when the initial FiO_2_ was 0.21–0.30 [44]. Two studies found a significant increase of markers of oxidative stress in preterm infants resuscitated with 90–100 % oxygen compared to 21 or 30 % [45, 46]. These findings indicate a possible mechanism how supplemental oxygen contributes to lung injury of preterm infants in the context of prenatal abnormalities, variables like positive pressure ventilation during transition and perinatal resuscitation and postnatal insults [47].

Therefore, current guidelines recommend using an initial FiO_2_ of 0.21–0.30 and to subsequently titrate FiO_2_ according to the infant’s SpO_2_ measured by pulse oximetry in order to avoid hyperoxia [48–50]. SpO_2_ measurement in preterm infants within the first minutes of life is feasible [51], and it is supposed to replace color as the traditional parameter for oxygenation [49]. However, aiming at variable SpO_2_ target values within the first 10 min of life is difficult, and large deviations from SpO_2_ targets during resuscitation of preterm infants have been observed in clinical studies [52], suggesting that manual FiO_2_ control in the delivery room is inadequate. A possible solution is the use of automated closed loop FiO_2_ control, which has been proven to efficiently keep infants within a predefined SpO_2_ target in the NICU, using various modes of ventilation, and using different algorithms (as reviewed in [53]). Although automated FiO_2_ control has not yet been tested in the delivery room setting in clinical trials [54], we could show in a lamb model of preterm respiratory distress syndrome that closed-loop FiO_2_ control is feasible during the transition after birth and during surfactant replacement therapy [55]. Moreover, automated FiO_2_ control during transition in the first 15 min of life resulted in less hyperoxia in our model [55]. Automated FiO_2_ control might therefore become a key element in balancing oxygen supplementation and in avoiding complications associated with early oxygen over- or underexposure.

## Mechanical ventilation as first or second hit

Oxygen therapy in the delivery room is regularly combined with manual inflations (“bagging”), ventilatory support with continuous positive airway pressure (CPAP), or mechanical ventilation. “Opening” the liquid-filled lung directly after birth in order to increase inspiratory volume and functional residual capacity (FRC) is a prerequisite for sufficient gas exchange. However, this early intervention can have lasting effects on the preterm lung. Experimentally, bagging of preterm lambs compromised the beneficial effect of surfactant replacement therapy [56]. Sustained lung inflation (SLI) increased FRC [57] but caused a modest increase of proinflammatory cytokines in the lungs of preterm lambs [58]. In a recent clinical trial, SLI did not decrease the occurrence of BPD in preterm infants born between 25 and 28 weeks and 6 days compared to a control group [59]. In this study, the need for mechanical ventilation within the first 3 days of life was decreased but not the overall need for respiratory support [59]. Experimentally, mechanical ventilation of preterm lambs increased inflammation and impaired developmental signaling in the lungs [60, 61]. However, mechanical ventilation might interact with prenatal factors. Prolonged mechanical ventilation increased the risk of BPD in a clinical study, and this effect was stronger when chorioamnionitis was present [11]. In contrast, antenatal betamethasone decreased lung injury but not lung inflammation in a preterm lamb model of resuscitation with escalating tidal volumes [62]. Avoidance of mechanical ventilation can be reached by utilizing CPAP with [63] or without [64] surfactant replacement therapy. Recently published data from the German neonatal network confirmed that surfactant replacement therapy in spontaneously breathing infants was associated with lower rates of mechanical ventilation and BPD [65]. Understanding the interaction between respiratory support and prenatally acquired preconditions might further help to minimize stress in the preterm lung.

## Caffeine—early and late effects on the lung

In the context of hypoxia, apnea of prematurity is widely recognized as a key problem in infants born prematurely. It has been successfully treated in the last three decades with methylxanthines, especially caffeine [66]. Caffeine is used both prophylactically and therapeutically, and a third indication is weaning from an endotracheal tube [67].

Although earlier trials had raised concerns about unwanted side effects like increased oxygen consumption and impaired weight gain [68], recent clinical trials showed impressive short-term and long-term beneficial effects of caffeine treatment in preterm infants [69]. In the Caffeine for Apnea of Prematurity (CAP) trial, the duration of positive pressure ventilation was shortened and supplemental oxygen could be stopped earlier in VLBW infants receiving caffeine instead of placebo as secondary outcome [67]. In the caffeine group, removal of endotracheal tube was possible at an earlier gestational age, and the need for postnatal steroids was significantly lower [67]. In line with these findings, a Cochrane review described less failure of extubation in infants receiving prophylactic methylxanthines (odds ratio 0.48, 95 % CI, 0.32–0.71) [70].

More interestingly, the CAP trial could show that caffeine reduced BPD, defined as need for supplemental oxygen at 36 weeks corrected gestational age, from 47 to 36 % [67]. This effect is presumably linked to the shortened duration of positive pressure respiratory support. However, a recent retrospective study revealed a strong correlation between high serum levels of caffeine and a decreased incidence of BPD in infants born ≤29 weeks GA [71]. These findings might result from a dose dependency of the beneficial effects of caffeine on lung function parameters and respiratory muscle strength [72]. Alternatively, preventive effect of caffeine for BPD might be linked to the anti-inflammatory effects on cytokine profiles of preterm babies which have been described recently [73], opening a promising field for future research.

Moreover, data from both the CAP trial and from retrospective cohort studies indicate how important timing of the start of caffeine therapy might be. In a subgroup analysis of the CAP trial, infants in whom caffeine therapy was initiated early, i.e., <3 days of age, had a significantly lower postmenstrual age at last endotracheal intubation and last positive pressure ventilation [74]. This suggests a possible mechanism for the decrease in BPD rates in infants receiving caffeine <3 days of age in two retrospective studies probably through less mechanical ventilation [69, 75]. However, early respiratory improvement might also be linked to additional therapeutic effects of caffeine. Caffeine is a known inhibitor of phosphodiesterase, and the consecutive bronchodilation by an increase of cyclic AMP might support the infants’ respiration [76]. In addition, experimental data suggest that caffeine amplifies the positive effect of prenatal glucocorticosteroids on surfactant-protein B expression, indicating a maturational effect of caffeine on the preterm lung [77]. In vitro, an additive effect on both transcription and translation of SP-B was shown [78]. This effect was confirmed in in vivo studies in spontaneously breathing preterm lambs born to ewes that received glucocorticoids. The preterm lambs received immediately after birth intravenous caffeine citrate and were maintained on CPAP. At the end of the study, the secreted SP-B in the bronchoalveolar lavage was several fold higher than in controls without caffeine [77]. However, although these findings suggest caffeine administration within the first hours of life or even in the delivery room as useful, the results of currently ongoing clinical trials [79] are needed to develop future recommendations.

## Pharmacological support of lung recovery and development—vitamin A

Drugs for postnatal modulation of lung injury have been extensively studied in the past. One of the most promising substances is vitamin A. Vitamin A is crucial for fetal lung development and maturation and prerequisite for adequate lung development [80, 81]. In preterm infants, vitamin A availability is lower than in term neonates [82]. Clinically, vitamin A supplementation reduces mortality and oxygen requirement at 36 weeks and is therefore considered as a promising pharmacological intervention in BPD prevention [83]. Ongoing clinical trials try to increase availability of this therapy by testing alternative modes of delivery [84]. In animal models, various mechanisms of lung protection by vitamin A as decreased lung fibrosis and increased lung elastin expression have been described [85, 86]. However, vitamin A is another example how therapeutic interventions depend on prenatal conditions. In a sheep model, intraamniotic exposure to inflammation reduced vitamin A in the lung [87], indicating that therapeutic benefit depends on the presence or absence of prenatal inflammation.

## Summary

The developing lung of the preterm infant is pre-, peri-, and postnatally exposed to different stress factors, and their impact depends on interaction between different insults and interventions. Prenatal exposure to chorioamnionitis preconditions the lung to postnatal stressors, by, e.g., immunological compromise and early disturbance of pulmonary developmental pathways. Understanding the interaction between two or more “hits” is a prerequisite for understanding mechanisms of permanent lung injury in preterm infants and for individualization of therapeutic interventions in order to promote recovery from the stressors. There is, e.g., evidence that maternal glucocorticoids should be given to all women at risk of impending preterm birth [18], even in the presence of clinical chorioamnionitis. Experimentally, the timing of steroid treatment in relation to the onset or the already existing chorioamnionitis made a difference. Information on the exposure of the baby to chorioamnionitis may therefore be of interest for clinical decision-making. Since chorioamnionitis is clinically silent in most instances, and histologic analysis of the placenta takes considerably long, a prediction model based on clinical parameters which are available upon delivery might support clinical decision-making [88]. Beside inflammation, intrauterine growth restriction (IUGR) has been linked to fetal lung injury and poor development [89], and infants suffering from IUGR had an increased BPD risk in a cohort study [90]. The genetic background might be another factor priming the lung towards temporary or permanent lung injury [91]. Therefore, detailed knowledge of the prenatal situation is absolutely essential to predict postnatal lung development.

Consequently, postnatal interventions need to be tailored individually to help the lung recover from early stress without causing more interventional stress than absolutely necessary. These might include early medication and oxygen treatment as discussed above but also other factors like adequate functional residual capacity (FRC) [92] and perinatal procedures like delayed cord clamping [93] and less invasive surfactant therapy [65] or the choice of a surfactant resistant to inactivation [94]. The mechanisms behind the influence of nutrition like the positive effect of exclusive breast feeding on BPD incidence [95] need to be further elucidated, and the full potential of known pharmacologic interventions like vitamin A supplementation needs to be explored. The knowledge of the combined effects of prenatal situation and postnatal interventions can help to further optimize potentially stressful therapeutic interventions and support lung recovery of preterm infants based on biology and increasing clinical evidence.
